# What is the point: will screening mammography save my life?

**DOI:** 10.1186/1472-6947-9-18

**Published:** 2009-04-02

**Authors:** John D Keen, James E Keen

**Affiliations:** 1 Department of Radiology, John H Stroger Jr Hospital of Cook County, 1901 West Harrison Street, Chicago, IL, 60612-9985, USA; 2 Department of Veterinary and Biomedical Sciences, University of Nebraska, PO Box 148, Clay Center, NE, 68933, USA

## Abstract

**Background:**

We analyzed the claim "mammography saves lives" by calculating the life-saving absolute benefit of screening mammography in reducing breast cancer mortality in women ages 40 to 65.

**Methods:**

To calculate the absolute benefit, we first estimated the screen-free absolute death risk from breast cancer by adjusting the Surveillance, Epidemiology and End Results Program 15-year cumulative breast cancer mortality to account for the separate effects of screening mammography and improved therapy. We calculated the absolute risk reduction (reduction in absolute death risk), the number needed to screen assuming repeated screening, and the survival percentages without and with screening. We varied the relative risk reduction from 10%–30% based on the randomized trials of screening mammography. We developed additional variations of the absolute risk reduction for a screening intervention, including the average benefit of a single screen, as well as the life-saving proportion among patients with earlier cancer detection.

**Results:**

Because the screen-free absolute death risk is approximately 1% overall but rises with age, the relative risk reduction from repeated screening mammography is about 100 times the absolute risk reduction between the starting ages of 50 and 60. Assuming a base case 20% relative risk reduction, repeated screening starting at age 50 saves about 1.8 (overall range, 0.9–2.7) lives over 15 years for every 1000 women screened. The number needed to screen repeatedly is 1000/1.8, or 570. The survival percentage is 99.12% without and 99.29% with screening. The average benefit of a single screening mammogram is 0.034%, or 2970 women must be screened once to save one life. Mammography saves 4.3% of screen-detectable cancer patients' lives starting at age 50. This means 23 cancers must be found starting at age 50, or 27 cancers at age 40 and 21 cancers at age 65, to save one life.

**Conclusion:**

The life-saving absolute benefit of screening mammography increases with age as the absolute death risk increases. The number of events needed to save one life varies depending on the prospective screening subset or reference class. Less than 5% of women with screen-detectable cancers have their lives saved.

## Background

Under ideal conditions, a woman participates in screening mammography after deciding that the potential benefit (increasing the length and quality of her life), considering the limitations, outweighs the expected harms and opportunity cost (time and money) [1]. Unrealistic expectations may influence a woman's decision. Regarding the potential benefit of screening mammography, two studies showed that over 90% of women think "early detection saves lives" [2], and a woman with screen-detected cancer "may have benefited" from mammography [3]. A woman may turn to her doctor for advice, but there is not universal agreement among physicians about the wisdom of screening mammography, especially in younger women ages 40 to 50 more susceptible to radiation-induced cancer [4]. The 2002 U.S. Preventive Services Task Force concluded that clinicians should stress that the benefit/harm ratio of screening improves with age [5]. The American College of Physicians recently advocated further research into the net benefits and harms of screening mammography in women ages 40 to 50 [6].

Furthermore, the components of informed medical decision-making regarding screening are not universally accepted [7-9]. For example, the relative mortality risk reduction (which makes screening sound more attractive) [10] was emphasized in one recent review of the screening mammography trials [11]. Many authors insist that the absolute rather than relative risk reduction is the most accurate way to describe a screening intervention [12,13]. Women are interested in knowing the absolute benefit before their first or baseline mammography exam. In one survey, 73% of women wanted to know the chance that their lives "will be prolonged by getting a mammogram" [14]. However, only 23% of these women were aware of the absolute benefit before initiating screening, and only 2% had a complete discussion of screening with their physicians [15]. The National Cancer Institute comprehensive web site provides only limited information, including the optimistic declaration "Absolute benefit is approximately 1% overall but depends on inherent breast cancer risk, which rises with age" [16].

Because popular claims such as "All women of the appropriate age should be screened" are often linked with the mantra "good quality screening mammography saves lives" [17], we decided to analyze how often this life-saving event occurs. Our goal was to help women and physicians understand the stated purpose of mammography. Ultimately, this knowledge is necessary to support consumer-oriented informed medical decision-making. We calculated the life-saving absolute benefit of screening mammography as a continuous function of age in the traditional format of the absolute risk reduction from repeated screening. To help women answer the question "What do I gain from today's mammogram?" we also estimated the average benefit of a single screening mammogram. Finally, to answer the question "Did that screening mammogram really save my life?" we derived the life-saving proportions from screening among women who develop or have a mammography-detectable breast cancer.

## Methods

### Absolute benefit defined

Absolute risk is defined as the probability of a particular event occurring over a specified period [18], such as the chance that an average driver will be killed in an automobile accident over 1 year. Three mathematically related statistics can describe the absolute benefit from an intervention or treatment designed to reduce the absolute risk of an event. For example, the relative risk reduction (RRR) might be the percentage reduction in non-fatal automobile accident injuries over 1 year in a group of drivers with an intervention of side airbags compared with a control group without side airbags. For ease of understanding, we will call the absolute risk reduction the reduction in absolute risk. Therefore, the first statistic is reduction in absolute risk = RRR * absolute injury risk, which can be presented as a frequency or as a percentage. Assuming hypothetically that the RRR of having side airbags is 30%, and the 1-year automobile non-fatal absolute injury risk is 1.0% or 10/1000 for average drivers over 12,000 miles [19], then the reduction in absolute risk would be 3/1000 ((0.30 * 10) injuries/1000 drivers), or 0.3%.

The second statistic, the number needed to treat to prevent one injury, is simply the reciprocal of the frequency form of the reduction in absolute risk [20-22]. In our example, the number of automobiles with side airbags needed to prevent one injury would be 333 (1000/3). Finally, the third statistic is the event-free percentage, which is the complement of the absolute risk in the untreated and treated groups in percentage form [23]. In our example, the event-free percentage over 1 year in automobiles without side airbags would be 99.0% (1.0 – 10/1000), and with airbags 99.3% (1.0 – 7/1000). The gain in the event-free percentage is simply the reduction in absolute risk in percentage form.

When the event is a death, the intervention causes a life-saving absolute benefit that reduces an absolute death risk. The absolute risk reduction equals the relative *mortality *risk reduction multiplied by the absolute *death *risk. Therefore, the first statistic becomes the reduction in absolute death risk or RADR = RRR * absolute death risk. The second statistic is the number needed to treat to save one life, and the third statistic is the survival percentage. We also developed two additional statistics for the life-saving absolute benefit for a screening intervention. These variations include the average benefit from one screening exam (using airbags for a long trip), as well as the life-saving proportions among women who develop or have earlier detection of cancer (lives saved for those in traffic accidents). Therefore, the life-saving absolute benefit in regards to screening has five forms: the RADR, number needed to screen, and the survival percentage; as well as the average benefit and the life-saving proportions.

### Absolute death risk

The first step in calculating the life-saving absolute benefit is to estimate the absolute death risk from breast cancer. Returning to the automobile example, assume both restraint use (seat belts) and vehicle improvements (anti-lock brakes) contributed to a reduction in the annual automobile accident absolute death risk over the last 2 decades since the promotion of seat belt use. To calculate the absolute benefit of seat belts, RADR = RRR * *absolute death risk*. If we used only the 1985 higher death risk without accounting for vehicle improvements, we would overestimate the seat belt benefit. Likewise, using the 2005 lower death risk would underestimate the seat belt benefit. To get the true seat belt benefit, we need to estimate a new baseline automobile absolute death risk without seat belts but with vehicle improvements. We can derive the baseline risk by subtracting the benefit of vehicle improvements from the 1985 higher death risk.

For breast cancer, we need to subtract the benefit of improved breast cancer treatment from the prescreening absolute death risk to get a new screen-free absolute death risk of breast cancer. Fortunately, these data are now available. Based on computer modeling, Berry et al estimated that screening mammography has been responsible for 46% (range 28%–65%) of the 30 percent breast cancer mortality reduction from 1975–2000, with the remaining 54% (range 35%–72%) due to therapy [24].

Fletcher and Harris previously used the cumulative probability of breast cancer death to present absolute death risk [1,25]. We obtained estimates of the cumulative probability of dying from DCIS and invasive breast cancer over 15 years by using the Surveillance, Epidemiology and End Results (SEER) Program mortality data for both 1978–1980 (higher pre-screening era death risk) [26,27] and 2002–2004 (lower current death and development risk) [26,28]. The 15-year period matches the median follow-up from analyses of the breast cancer screening randomized controlled trials (RCT) [11,29]. We included women ages 40 to 65 in 5-year age increments.

Therefore, to estimate the current therapy but screen-free absolute death risk, we first needed to calculate a risk difference = H - L, with H = higher 1980 prescreen death risk and L = lower 2004 current death risk. We started with the SEER 1978–1980 death risk per 1000 women including both DCIS and invasive cancer, e.g. for age 40 H = 5.95 (95% confidence interval (CI) 5.71–6.19). We subtracted the 2002–2004 death risk, L = 3.73 (95% CI 3.64–3.81), to obtain the risk difference or mortality improvement, H - L = 2.22. The 95% CI is 1.96–2.48 using Monte Carlo resampling. We multiplied the risk difference by the fraction attributable to therapy or 0.54. We then subtracted this therapy benefit (0.54 * 2.22 = 1.20) from the 1978–1980 death risk to obtain a new screen-free absolute death risk, e.g. 5.95 – 1.20 = 4.75 at age 40. We calculated the range (4.30–5.32) by using interval analysis.

### Reduction in absolute death risk

With the new screen-free absolute death risk from breast cancer, we can calculate the life-saving absolute benefit of screening knowing the RRR since RADR = RRR * absolute death risk. The risk ratio of death from the RCT of repeated screening mammography is the ratio of cumulative follow-up breast cancer deaths in the screened group to the deaths in the control group, adjusted by the person-years in each group. The complement of the risk ratio is the RRR of repeated screening [30]. Analyses of the screening trials estimate that the RRR is 16%–20% overall, 15% for women under 50 and 22% for women over 50 after 14 years of follow-up [11,29]. We considered RRR values from 10% to 30%, with a base case of 20%. Arguments can be made that this value should be lower (RCT issues) or higher (screening compliance) [31,32]. By multiplying this RRR by the screen-free absolute death risk, we estimated the cumulative RADR from repeated screening for women in the United States. We used the RADR to calculate the number needed to screen repeatedly (NNSR) and the survival percentages.

### Average benefit

The RRR from the analyses of the screening trials is due to women undergoing multiple or repeated screening mammograms over a median period of about 6 years. To estimate the number of mammogram examinations needed to save one life, the NNSR needs to be multiplied by the median average number of screening rounds of 4.25 (range 2–9) [29]. Tabar et al previously used this approach after analysis of one randomized trial; however, the authors did not obtain age-specific estimates [33]. The benefit of the first or baseline mammogram (prevalence screen) is at least twice the benefit of repeated or subsequent exams (incidence screen) in terms of cancer detection [11]. Therefore, if we count the baseline mammogram twice, the median number of screening rounds would be 5.25 (range 3–10). Now, we can assume that every single mammogram contributes equally to the benefit from repeated screening, so the benefit/mammogram would be independent of time. Therefore, the number of mammogram examinations needed to save one life would be equal to the number needed to screen once (NNSO) at some time during 6 years. The reciprocal of the NNSO is the average life-saving absolute benefit of one subsequent mammogram. The average benefit of the baseline mammogram would be twice that for a subsequent exam. We calculated the range for the RADR, NNSR and the number of mammogram examinations by using interval analysis.

### Life-saving proportions

We also estimated the fraction or proportion of women with life-saving mammography in the two following groups: those who will develop cancer, and those who can have their cancer detected by screening mammography. The numerator of the life-saving proportion (LSP) is the screen-free absolute death risk multiplied by the RRR. The denominator is the SEER 2002–2004 cumulative 15-year probability of developing cancer, multiplied by the chance of having that cancer detected (mammography sensitivity), or the cumulative cancer detection rate (CCDR). Therefore, the LSP = RADR/CCDR. We assumed a cumulative sensitivity of 80% for repeated mammography over the period of screening [16]. The reciprocal of the LSP is the number of cancers needed to develop or be detected (NND) to save one life. The maximum LSP would occur with the highest possible screening benefit by assuming no therapy improvements, so we calculated this effect for a sensitivity analysis.

### Life-saving percentages

We can summarize our results by converting the life-saving absolute benefit of mammography in the various screening subsets that we have analyzed into the life-saving percentage of events in each subset. These prospective subsets or reference classes [34] include women screened once, women screened repeatedly, women who develop cancer, women who have cancer detectable, and women who die of cancer. These percentages answer the question, "Will screening mammography save my life?" We also presented our results in augmented and frequency formats to increase comprehension [35].

To simplify our results, we also generated scatter plots of death risk and development risk versus age in Excel (Microsoft, Redmond WA). We determined the best-fitting trend line by simple linear regression as defined by the highest coefficient of variation (R^2^).

## Results

### Absolute death risk

**Figure 1:**
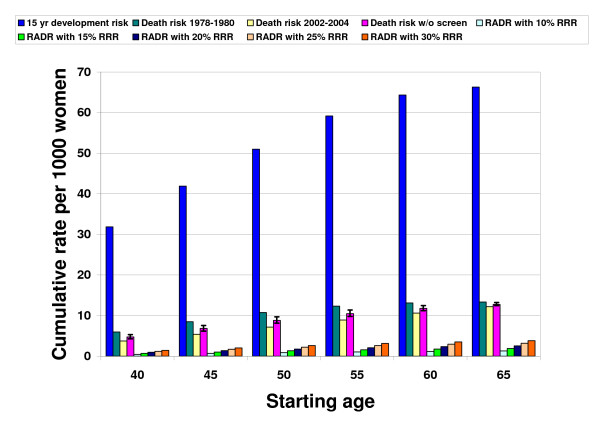
**The life-saving absolute benefit or reduction in absolute death risk from repeated screening mammography according to age**. Data are from the Surveillance, Epidemiology, and End Results (SEER) Program including women ages 40 to 65 in five-year age increments. The first column in each age group shows the 2002–2004 cumulative 15-year development risk for breast cancer for average women. The 1978–1980 cumulative 15-year absolute death risk from breast cancer (2^nd ^column) is higher than the 2002–2004 death risk (3^rd ^column) due to screening effects and better therapy. We multiplied plausible values for relative risk reduction (RRR) of 10% to 30% from repeated screening by the screen-free absolute death risk (4^th ^column) to achieve estimates of the reduction in absolute death risk (RADR) from repeated screening (5^th ^to 9^th ^columns). The RADR is the same as the life-saving absolute benefit or absolute risk reduction. Starting at age 50 and 20% RRR, the RADR is 1.8/1000. The numerator is the same as lives saved per 1000 women screened. Between the starting ages of 40 and 55, the life-saving absolute benefit from mammography more than doubles, corresponding to the increased death risk. Between the starting ages of 50 and 60, the RRR from repeated screening is about 100 times the absolute risk reduction since the screen-free absolute death risk is approximately 1%.

Figure 1 shows the age dependence of the development risk and the screen-free absolute death risk from breast cancer. The development risk = [-0.040AGE^2 ^+ 5.63AGE - 129.6]/1000, R^2 ^= .99. The absolute death risk varies between 0.475% at age 40 to 1.274% at age 65. The best-fit equation = [-0.006AGE^2 ^+ 0.96AGE - 23.9]/1000, R^2 ^= .99. Between the starting ages of 40 and 55 (1.05%), the absolute death risk over 15 years more than doubles. Between the ages of 50 (0.883%) and 60 (1.177%), the absolute death risk is about 1%.

### Reduction in absolute death risk

Figure 1 shows the RADR of repeated screening mammography at different levels of RRR. For perspective, the first column in each age group shows the 15-year development risk. For example, at 20% RRR and starting at age 50, repeated screening averts 1.8 breast cancer deaths (range 1.6–1.9) over 15 years for every 1000 women screened. The RADR varies from 2.4/1000 (2.3 to 2.5) at age 60, to 1.0/1000 (0.9 to 1.1) at age 40. The age/RRR extremes of the RADR are 0.5/1000 at age 40/10% RRR, to 3.8/1000 at age 65/30% RRR, a ratio of 7.6. At the starting age of 53, the RRR is 100 times the RADR or absolute risk reduction, since the screen-free absolute death risk is 1% (RADR/0.01 = RRR).

**Figure 2:**
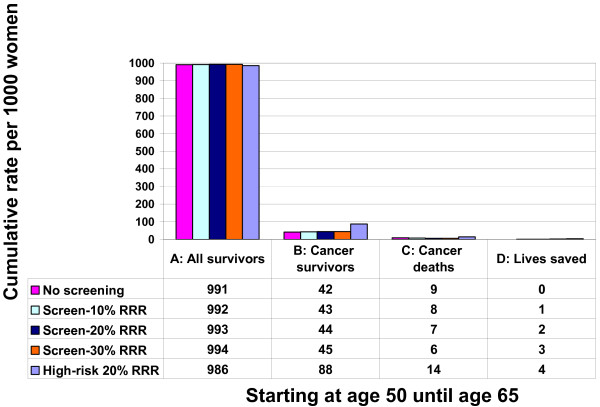
**Frequency of survival for all women and women with cancer, deaths from breast cancer and lives saved from repeated screening mammography**. Starting at age 50, 51 out of 1000 women will develop breast cancer over 15 years (Figure 1). In terms of natural frequencies, the 1000 healthy average-risk women are the reference class. Figure 2 Group A shows 991 out of 1000 women will survive (not die from) breast cancer by age 65 without screening. Group B shows that 42 of the 51 breast cancer patients survive (positive framing) without screening. Therefore, nine women die (negative framing) without screening (Group C), and screening saves no lives (Group D). Assuming a 20% relative risk reduction (RRR) from repeated screening (row 3), mammography prevents two of nine deaths through earlier treatment, leaving seven cancer deaths. This means 44 of the 51 cancer patients and 993 of 1000 women will survive breast cancer.

**Figure 3:**
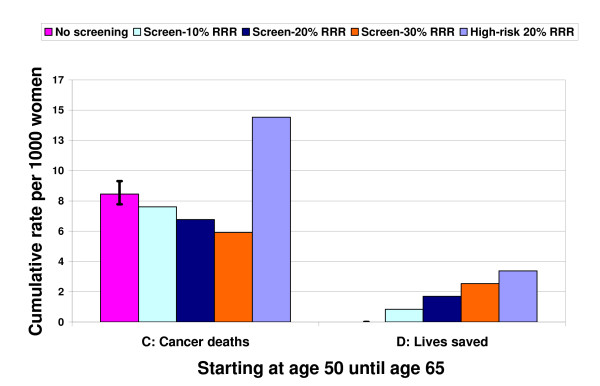
**Deaths from breast cancer and lives saved from repeated screening mammography**. Figure 3 is a magnification view of Groups C and D from Figure 2. Compared to Figure 1, the nine deaths without screening are the same as the screen-free absolute death risk, while the lives saved are the same as the corresponding reduction in absolute death risk. Four out of 1000 high-risk (double the average risk) women will have their lives saved assuming a 20% relative risk reduction (RRR).

The reciprocal of the RADR is the NNSR, which is 1050 (940–1160) at age 40, 570 (520–620) at age 50, and 430 (400–450) at age 60. This means that if screening starts at age 50, repeated screening of 570 mostly healthy women saves one life among the many women who develop cancer over 15 years. The survival percentage shown in Table 1 is 100 times the complement of the absolute death risk in the unscreened and screened groups. The survival percentage at age 50 without screening means that 99.12% of women do not die from breast cancer over 15 years, which improves to 99.29% at a 20% RRR with screening. The gain in survival percentage is the RADR in percentage form (0.177%). Figures 2 and 3 present the survival frequencies starting at age 50 given selected RRR for all women (from Table 1) and for women who develop breast cancer, as well as cancer deaths and lives saved (from Figure 1) using the reference class of 1000 average women. We also included the reference class of 1000 high-risk women, defined as having twice the average cancer development and death risk.

**Table 1:** Survival percentage: women not dying from breast cancer without and with repeated screening over 15 years.

Age	No screening*	**10% RRR**^**†**^	20% RRR	30% RRR
		**with screening**	**with screening**	**with screening**
				
**40**	99.52%	99.57%	99.62%	99.67%
**45**	99.32%	99.39%	99.45%	99.52%
**50**	99.12%	99.21%	99.29%	99.38%
**55**	98.95%	99.06%	99.16%	99.27%
**60**	98.82%	98.94%	99.06%	99.18%
**65**	98.73%	98.85%	98.98%	99.11%

* Survival percentage without screening is 100 times the complement of the screen-free absolute death risk from Figure 1.

^† ^Survival percentage with screening is the same as the reduction in absolute death risk in percentage form from Figure 1 added to column 2 of this table.

### Average benefit

Table 2 shows the number of mammogram examinations needed to save one life. Assuming equivalent benefit from all mammograms, this becomes equal to the NNSO. Starting at age 50 and 20% RRR, screening 2970 women once (range 1540–6160) will save one life over the next 15 years. This number is 5.25 times the NNSR of 570, or 5.25/RADR. The average benefit from one subsequent mammogram would be 1/2970, or 0.034% chance of a life saved. At age 40, assuming a 10% RRR, the NNSO increases to 11050, and at age 60 at 30% RRR, decreases to 1490.

**Table 2:** Number of mammogram examinations needed to prevent one death.*

Age	**10% RRR**^**†**^	15% RRR	20% RRR	**Range 20%**^**‡**^	25% RRR	30% RRR
**40**	11050	7370	5530	2820–11630	4420	3680
**45**	7700	5130	3850	1980–8070	3080	2570
**50**	5950	3970	2970	1540–6160	2380	1980
**55**	5000	3330	2500	1320–5100	2000	1670
**60**	4460	2970	2230	1200–4450	1780	1490
**65**	4120	2750	2060	1130–4020	1650	1370

* Number of examinations is 5.25 times the number need to screen repeatedly (NNSR), which is the reciprocal of the reduction in absolute death risk (RADR) in Figure 1. The number needed to screen once (NNSO) is equivalent to the number of examinations if the benefit is the same for all mammograms. The average benefit of a subsequent screen is the inverse of the NNSO.

^† ^The 5.25 median screens from the trials are responsible for the relative risk reduction (RRR) for repeated screening. We assumed the baseline exam has twice the benefit of a subsequent exam [11,29,33].

^‡ ^The range is calculated by using interval analysis.

### Life-saving proportions

Finally, Table 3 shows the proportion of women with screen-detectable cancers that have their lives saved over 15 years through earlier treatment because of mammography. The LSP ranges from 1.9% to 7.2% depending on age and RRR. Assuming a 20% RRR starting at age 50, the life-saving proportion is 4.3%, which falls to 3.7% at age 40 and increases to 4.6% at age 60. Therefore, the NND to save one life starting at age 50 is the reciprocal of 4.3%, or 23.

**Table 3:** Life-saving proportion: women with screen-detected cancers that have their lives saved by mammography.

Age	Development risk/1000*	**CCDR/1000**^**†**^	**Death risk/1000**^**‡**^	10% **RRR**^**§**^	15% RRR	20% RRR	20% **NND**^**∥**^	25% RRR	30% RRR	20% RRR **No Rx**^**¶**^
				**%**	**%**	**%**	**%**	**%**	**%**	**%**
										
**40**	31.9	25.5	4.8	1.9	2.8	3.7	27	4.7	5.6	4.7
**45**	41.9	33.5	6.8	2.0	3.0	4.1	25	5.1	6.1	5.1
**50**	51.0	40.8	8.8	2.2	3.2	4.3	23	5.4	6.5	5.3
**55**	59.2	47.3	10.5	2.2	3.3	4.4	23	5.5	6.7	5.2
**60**	64.4	51.5	11.8	2.3	3.4	4.6	22	5.7	6.9	5.1
**65**	66.3	53.0	12.7	2.4	3.6	4.8	21	6.0	7.2	5.0

* Cumulative 15-year development risk for breast cancer for average women, from Figure 1 [28].

^† ^Cumulative cancer detection rate (CCDR) over 15 years assumes a cumulative sensitivity of 80% from repeated screening.

^‡ ^Breast cancer screen-free absolute death risk is from Figure 1.

^§ ^The proportion is the screen-free absolute death risk multiplied by the relative risk reduction (RRR), and then divided by the CCDR.

^∥ ^Number of cancers needed to be detected (NND) is the reciprocal of the life-saving proportion.

^¶ ^Assumes maximum prescreening era (1978–1980) 15-year absolute death risk, with no adjustment for improved therapy.

### Life-saving percentages

**Figure 4:**
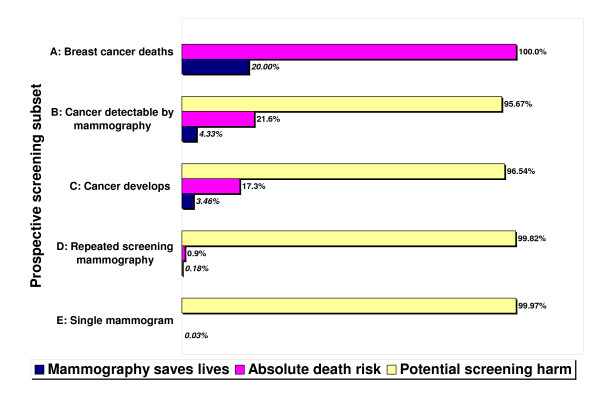
**Will screening mammography save my life?**. The answer depends on the reference class. The life-saving absolute benefit of mammography increases as the absolute death risk in the woman's prospective screening subset increases. For women starting at age 50 and assuming a 20% relative risk reduction (RRR), the chance that "mammography saves lives" appears as the bottom row for each of the five screening subsets. For instance, mammography saves 4.3% of screen-detectable cancer patients' lives (subset B). These life-saving percentages correspond to the RRR (A: Breast cancer deaths), life-saving proportions (B&C), reduction in absolute death risk (D: Repeated screening mammography – see Figure 1), and average benefit (E: Single mammogram). For a 20% RRR, the underlying absolute death risk (middle row) is five times the life-saving percentage. The number of events needed to save one life in each subset is the reciprocal of the life-saving percentage and increases from the smallest subset A (5) to the largest subset E (2970). Potential screening harm (top row, 100% minus bottom row) for women with cancer includes overdiagnosis, overtreatment, and delayed diagnosis. The potential harm for healthy women includes false-positive evaluations and biopsies, screening associated anxiety, and radiation-induced cancer.

**Figure 5:**
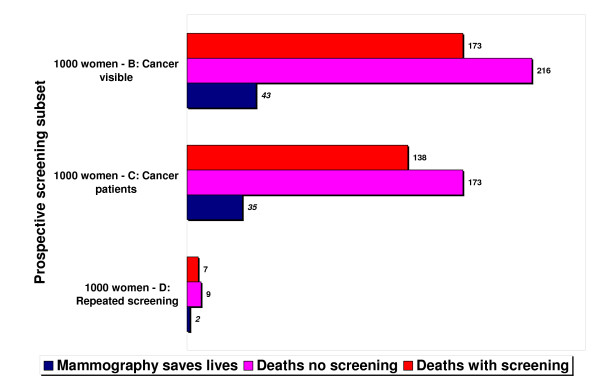
**Frequency of lives saved from screening mammography for 1000 women in three prospective screening subsets**. Figure 5 is a magnification view for subsets B, C, and D from Figure 4 but with 1000 women in each reference class. The frequency of breast cancer deaths without screening and the frequency of lives saved from mammography are equivalent to the percentages in Figure 4. The top row is the difference, or the breast cancer deaths despite screening in each subset. Compared to Figure 3, the nine deaths without screening and the seven cancer deaths and two lives saved with screening starting at age 50 and 20% relative risk reduction are equivalent.

Using Figure 1 column 1, if we assume a group of 2000 40-year-old women, without screening, about 10 (4.8 * 2 = 9.6) will die of breast cancer over the next 15 years. Assuming a low end 10% RRR for younger women instead of the 20% base case, screening saves one of these 10, a life-saving percentage of 10%. From the perspective of the 64 (31.9 * 2 = 63.8) of these 2000 women who will develop cancer over 15 years, about 51 (0.8 * 64 = 51) could have their cancer detected by screening. Table 3 column 5 shows that repeated mammography will save one of these 51 women, or a life-saving percentage of 1.9% (0.019 * 51 = 0.97), but 0% of the 13 (64-51) women whose cancer is not detectable. The RADR for all 2000 women undergoing repeated screening is 1/2000, or a life-saving percentage of 0.05%. Using Table 2 column 2, if all mammograms have equivalent benefit, then screening about 11050 women once will save one life, a life-saving percentage of 0.009%. By using the same methodology, Figure 4 summarizes how often "mammography saves lives" for the five prospective screening subsets. For the age 50 base case, screening mammography saves 20% of women who would die from cancer over 15 years. While four of the prospective subsets have known death risks, we would have to impute the death risk associated with the single mammograms. The base case ratio of life-saving absolute benefit high/low subsets is 130 (4.3/0.034). Figure 5 shows three subsets from Figure 4, each with a reference class having 1000 women.

## Discussion

We have tried to explain the purpose of mammography by answering the question: how often will screening mammography "save" a woman's life? We have shown that since the RADR = RRR * absolute death risk, the answer for women undergoing repeated screening increases with the age-related absolute death risk. For the age range 50 to 60, the screen-free absolute death risk for average women is approximately 1%, so the RRR from repeated screening is about 100 times the RADR or absolute risk reduction.

The life-saving absolute benefit also depends on the prospective screening subset or reference class to which a woman will belong. For women who will develop cancer, the absolute death risk increases, so the life-saving absolute benefit of mammography increases. Women who will die of cancer have an absolute death risk of 100%, so the life-saving absolute benefit is the maximum or the RRR. For the base case age 50 and assuming a 20% RRR, women with a screen-detectable cancer have a 4.3% chance of truth for the claim that "screening mammography saved my life."

We can express the life-saving absolute benefit using percentage or frequency forms. The low extreme is the average benefit from a single mammogram of 1/11050 screens (0.009% at age 40/10% RRR). The high extreme is the life-saving proportion for women with a screen-detectable cancer of 1/1370 (7.3% at age 65/30% RRR). For the base case 20% RRR, the life-saving absolute benefit of a single mammogram is 1/2970 screens, or 0.034%. For multiple or repeated screening, the RADR is 1.8/1000, so the life-saving percentage is 0.18%. Viewing these results from a different perspective of the number of events needed to save one life in each screening subset, the following values are equivalent: five women dying of breast cancer, 23 women with screen-detectable cancers, 29 women with cancers present, 570 women screened repeatedly, and 2970 women screened once. In other words, by subtracting the one life saved in each prospective screening subset, there is no life-saving absolute benefit for 22 women with screen-detectable cancers, or for 2969 women screened once.

### Prior analyses

Our findings are close to other estimates of absolute benefit from screening mammography, which will vary due to the RRR and the cumulative period used. For instance, the SEER 2002–2004 10-year cumulative risk of breast cancer death from age 40 to 50 is 0.194%, versus 0.373% or 3.73/1000 (Figure 1) from age 40 to 55 [28], for a ratio of 0.52. Harris estimated that over a decade of regular screening the following lives would be saved over 15 to 20 years: 1.2–1.8/1000 at age 40, 1.9–3.9/1000 at age 50, and 5.9/1000 at age 60 [25]. Fletcher et al estimated that assuming 10 years of annual screening, 2/1000 lives are saved over 20 years at age 40 (20% RRR) which increases to 4/1000 at age 50 (30% RRR) [1]. Barratt et al constructed a decision model of biannual screening for 10 years which predicts a RADR of 0.5/1000 at age 40 (23% RRR), and 1.9/1000 at age 50 and 3.0/1000 at age 60 (37% RRR) [36]. Schwartz et al estimated a 10 year absolute risk reduction of 0.8/1000 ages 40 to 49 (24% RRR), and 3/1000 ages 50 to 70 (33% RRR) [37]. Estimates of the NNSR from all the trials for women under age 50 given a 15% RRR are 1792 (95% CI 764–10540) or absolute risk reduction of 0.56/1000 (95% CI 0.09–1.31) which decreases to 838 (95% CI 494–1676) assuming a 22% RRR for women 50 and over [6,29].

Tabar et al found that the NNSR estimate from a single trial without any analysis of the effect of a woman's age was 465 (95% CI 324–819) over 20 years, given a 30% RRR, while the number of mammogram examinations was 1499 (95% CI 1046–2642) [33]. This estimate is lower than our value of 1980 (range 1030–4110) at age 50. If we adjust the absolute death risk to 10 years from 15 and we use a 17% RRR starting at age 40 [28], we calculate an absolute risk reduction of 0.4/1000 (0.95 * 0.52 * 0.85 = 0.42). This estimate matches the result from the recent single randomized screening trial limited to younger women under 50 and beginning at age 40 [38]. The overall RADR over 10 years recently calculated for all the RCT of screening mammography is 0.05% or 0.5/1000 [31].

### Mammography benefit

First, we have quantified how the life-saving absolute benefit of mammography gradually increases with age, which is important for younger women considering the age at which to begin screening [5,6]. For example, the RADR at age 65 is 2.7 times the RADR at age 40. Second, we have estimated the life-saving absolute benefit from a single mammogram. This statistic is useful because women make the decision to screen about 30 million times in the United States each year [39]. The average benefit reinforces the fact that the RRR from screening depends on multiple or repeated exams, and it eliminates the assumptions regarding the frequency and length of screening. Finally, we have challenged the popular perception that earlier detection through mammography helps most patients with screen-detected breast cancer [2,3].

Our analysis of "mammography saves lives" addresses a knowledge gap and may facilitate better information exchange before women initiate screening [15]. Many women consistently overestimate the true benefit of screening mammography, most importantly by confusing primary and secondary prevention. For instance, two recent surveys showed that over half of women think that mammography acts like a vaccine by preventing or reducing the risk of contracting breast cancer [40,41]. This misunderstanding is analogous to thinking that seat belts are like antilock brakes and can prevent traffic accidents as well as accident deaths. In a study almost a decade ago, women under 50 had a greatly inflated perception of the absolute risk (20 times) of dying from cancer over 10 years, and the absolute risk reduction (over 120 times) derived from screening. These findings were consistent with the perception that most breast cancer is rapidly fatal and that women falsely equate *diagnosis *risk with *death *risk [42]. This confusion is not surprising considering the one in eight (12.3%) cumulative development risk for breast cancer between the ages of 40 to 80 is rarely linked with the one in 50 (2.0%) cumulative death risk for breast cancer during the same time span [28]. SEER data in Figure 1 show the current 15-year cumulative diagnosis or development risk is about seven times the cumulative death risk, which implies a long average survival after diagnosis [43].

Consequently, women should have improved insight and reduced anxiety with a balanced presentation of the absolute benefit of mammography in terms of the diagnosis risk as well as the death risk [42]. Table 3 shows that more than 95% of the time, screening mammography will not save a screen-detectable cancer patient's life. This finding is counter-intuitive since "Women view breast cancer as a uniformly progressive disease rarely curable unless caught early" [2]. In reality, breast cancer is a heterogeneous disease that may be systemic from the start or never metastasize [44]. Yet the belief in the exaggerated efficacy of earlier detection is widespread: a recent survey of cancer screening attitudes found that 74% of the general population believes that finding cancer early saves lives most or all of the time [45]. Assuming 100% cumulative sensitivity of mammography, the upper limit of the LSP would be at most the RRR (10%–30%), and only if the death risk were equal to the development risk.

### Screening promotion

One reason for the public's confusion is that screening statistics can be difficult for even highly educated physicians to understand, and this innumeracy presents a roadblock to consumer insight about the true benefit of mammography [10]. Rather than address innumeracy and promote informed decision-making [46], most health organizations encourage high participation rates [2], rationalized in the interest of "public" health [47]. The recent single randomized screening mammography trial limited to younger women under 50 and beginning at age 40 found a statistically non-significant 17% RRR [38]. Despite this finding, the American Cancer Society still has a goal to increase screening mammography participation in women over 40 from 67% to 90% by 2010 [48]. Analysts also reveal a preference for screening uptake rather than insight when they express concern about any decrease in mammography utilization [49-51].

Figure 4 supports the analysis by Berry that screening mammography is a lottery [52]. Screening advocates indirectly acknowledge this analogy by focusing on the jackpot (mammography saves lives) rather than the average gain. One estimate of the average gain to a woman after a decade of screening is an extra week of life. This 1 week gain ignores substantial "private" psychological costs (including false-positive mammogram and biopsy-induced anxiety), which would reduce the average gain to between 2 and 3 days [53]. This adjusted gain is likely less than the total time lost by a woman in order to get the mammograms. Yet unlike a lottery ticket, there is no fine print discussing the long odds of saving a life on the back of screening reminder letters [54].

Besides the opportunity cost of time and money (buying the ticket), authorities acknowledge that screening mammography harms include a 30% increase in overdiagnosis and overtreatment, delayed diagnosis, and radiation-induced cancers [16,31,55]. One survey showed that physicians discuss these harms with women only 7% of the time before the baseline mammogram [15]. One reason may be that authors often ignore these harms in scientific articles about screening mammography [56]. By this time, editors, scientists and physicians should know and acknowledge the potential problems and inherent biases with screening [37,57-59].

Instead of providing professional even-handed advice, breast radiologists usually enthusiastically promote expensive new screening technologies developed by the medical imaging industry despite the drawbacks [8,17,60,61]. For example, digital mammography may have decreased sensitivity in older women (who are more likely to get cancer), as well as increased false-positive examinations and biopsies related to digital technology linkage with computer-aided diagnosis [62]. Magnetic resonance and ultrasound imaging both substantially increase recall rates, and neither technology has any proven mortality benefit [63,64]. Consumers need to keep in mind that fundamentally, cancer testing is a business [18,52]. Breast radiologists, equipment manufacturers [65], and advocacy groups [66] all have a conflict of interest due to financial incentives. These incentives create the temptation to exaggerate the benefits and dismiss the harms of screening for breast cancer when advertising to the public. As one proponent of informed decision-making observes, excessive fear of breast cancer helps certain interest groups but is not in the best interest of women [10].

### Consumer education

Voluntary informed participation requires that women and their physicians understand that earlier detection and treatment of breast cancer through screening mammography (and possibly through other imaging technology) certainly saves *some *lives, but includes significant harms to many women with and without cancer. Figure 1 shows how the absolute death risk increases substantially with age, and that many women die *with *breast cancer, not *from *breast cancer. Education campaigns for breast cancer awareness that want to promote insight rather than uptake should stress the death risk from breast cancer along with the development risk. For example, Figure 5 shows that given 1000 breast cancer patients and a 173/1000 death risk without screening, 827/1000 cancer patients are "cured" or live a long time without mammography.

We have shown that between the starting ages of 50 and 60, the RADR or absolute risk reduction is only about 1% of the RRR. In order to address the problem of innumeracy, health organizations should advertise the absolute death risk and the absolute benefits along with the corresponding RRR [67,68]. Figures 4 and 5 show that if women misapply the RRR by using a reference class besides breast cancer deaths, they will overestimate the absolute benefit of screening [34]. To avoid manipulation and to foster insight, health organizations should frame the risk or benefit by using both positive terms (absence of disease or survival) and negative terms (presence of disease or death) [46,69]. For example, Figure 5 shows that screening would save 35 but would not save 138 of the 173 women who die from breast cancer. From Table 1, younger women at age 40 have a 99.5% chance of *not *dying from breast cancer before age 55, which improves to 99.6% with regular screening (a gain in survival percentage of 0.1%, not 1.0%) [16].

Analysts can best communicate statistical information by using natural frequencies (event counts using one reference class) or clarifying the reference class [34]. For instance, assuming 2970 single screening mammograms are required to save one life starting at age 50 (Table 2), this means that about 370 (12.4/100) women will be recalled for additional testing with associated psychological distress, and about 62 (2.1/100) biopsies will be required [70]. For repeated screening and using a 16% RRR [29] (Table 3), screening mammography prevents three to four deaths over 15 years in 100 screen-detected cancer patients under age 65. Approximately 30 of the remaining women will experience overdiagnosis and overtreatment, including mastectomies and radiation treatment that can cause heart disease [31,71].

A comparison with equivalent risks will help put the death risk from breast cancer in perspective. For example, the cumulative death risk from lung cancer is 0.93% or 9.3/1000 between the ages of 50 and 65 [28], similar to the death risk from breast cancer of 0.88% or 8.8/1000. The absolute risk of dying as a vehicle occupant in an automobile accident projected over 15 years driving 12,000 miles a year is 0.205% or 2.05/1000 [19]; this death risk is equal to our base case life-saving absolute benefit of screening mammography of 1.8/1000 if a woman drives 10,300 miles a year. The lifetime development risk for coronary heart disease for women over age 40 of 25%–32% is at least twice the development risk for breast cancer [72].

### Limitations

From the RCT survival curves, analysts cannot tell if the mortality reduction associated with screening mammography is due to either a "cure" or "reduction in hazard". We have assumed that a "life saved" means screening helps cure one woman with breast cancer who would have otherwise died from the disease without screening. The end result from a cure is that a woman lives a normal life span beyond the point of expected death from breast cancer. However, all women with cancer may theoretically benefit from screening mammography through slowing the disease and therefore slightly prolonging their lives. These women would experience life extension but would still die from breast cancer. The source of the survival benefit from screening mammography may lie somewhere in between individual life saving and multiple smaller life extensions, so the distribution of women actually benefiting in some way from screening (although the total effect is the same) is not known [73].

In economic theory, the marginal cost of a defensive measure taken to reduce the risk to health and life must be less than the marginal benefit from the reduced probability of disease and death, or the action should not be undertaken [74]. Therefore, deriving the equivalence of "saving a life" in terms of a present-value quality-adjusted amount of life saved per women screened would simplify the expected benefit from screening and help women make better decisions [75]. The marginal cost per women screened includes the expected opportunity cost (time, travel, and resource costs) and the expected harms (including anxiety and overtreatment). Multiple factors will influence this economic analysis, including a woman's attitude toward risk. Younger women have longer normal lives than older women and more potential years to save through screening mammography. On the other hand, older women have more potential cancer to find through the increasing incidence of breast cancer with age [62]. Furthermore, there may be a benefit of screening other than mortality reduction through reduced morbidity from less aggressive breast cancer treatment. However, women must weigh this possible benefit against the effect of screening-induced overdiagnosis and overtreatment [55].

Our study is limited because the screening effect on the absolute risk of death from any cause may be more meaningful than the screening effect on breast cancer mortality from the RCT [18,76]. Our base case assumption of a 20% RRR due to screening may be optimistic and is probably closer to 15% [29,31]. Finally, further analysis of the relative contribution of the baseline versus the subsequent mammogram in reducing breast cancer mortality may produce a better estimate of the average benefit of a single mammogram.

## Conclusion

We have shown how the life-saving absolute benefit of screening mammography gradually increases with age as the screen-free absolute death risk increases, since RADR = RRR * absolute death risk. For 50- to 60-year-old women, the screen-free absolute death risk over 15 years is approximately 1%, so the RRR for repeated screening mammography is about 100 times the RADR or absolute risk reduction. The chance that "mammography saves lives" depends on the absolute death risk in the prospective screening subset or reference class to which a woman belongs. For a woman in the screening subset of mammography-detectable cancers, there is a less than 5% chance that a mammogram will save her life. By comparing screening mammography's life-saving absolute benefit with its expected harms and her opportunity cost, a well-informed woman along with her physician can make a reasonable decision to screen or not to screen for breast cancer.

## Abbreviations

*CCDR*: Cumulative cancer detection rate; *CI*: Confidence interval; *LSP*: Life-saving proportion, or the reciprocal of the NND; *NND*: Number of cancers needed to develop or be detected; *NNSO*: Number needed to screen once, or the reciprocal of the average benefit; *NNSR*: Number needed to screen repeatedly, or the reciprocal of the RADR; *RADR*: Reduction in absolute death risk, or RRR times the absolute death risk; *RCT*: Randomized controlled trials of screening mammography; *RRR*: Relative risk reduction; *SEER*: Surveillance, Epidemiology and End Results.

## Competing interests

The authors declare that they have no competing interests.

## Authors' contributions

JDK conceived the study, developed the methodology, and drafted the manuscript. JEK performed the statistical analysis. Both JDK and JEK analyzed the results, and revised and approved the final manuscript.

## Pre-publication history

The pre-publication history for this paper can be accessed here:

http://www.biomedcentral.com/1472-6947/9/18/prepub
